# *Cicer turcicum:* A New *Cicer* Species and Its Potential to Improve Chickpea

**DOI:** 10.3389/fpls.2021.662891

**Published:** 2021-04-15

**Authors:** Cengiz Toker, Jens Berger, Tuba Eker, Duygu Sari, Hatice Sari, Ramazan Suleyman Gokturk, Abdullah Kahraman, Bilal Aydin, Eric J. von Wettberg

**Affiliations:** ^1^Department of Field Crops, Akdeniz University, Antalya, Turkey; ^2^CSIRO Agriculture and Food, Wembley, WA, Australia; ^3^Department of Biology, Akdeniz University, Antalya, Turkey; ^4^Department of Field Crops, Harran University, Şanlıurfa, Turkey; ^5^Department of Plant and Soil Science and Gund Institute for Environment, University of Vermont, Burlington, VT, United States

**Keywords:** *Cicer*, new species, genetic resources, heat tolerance, bruchid resistance

## Abstract

Genetic resources of the genus *Cicer* L. are not only limited when compared to other important food legumes and major cereal crops but also, they include several endemic species with endangered status based on the criteria of the International Union for Conservation of Nature. The chief threats to endemic and endangered *Cicer* species are over-grazing and habitat change in their natural environments driven by climate changes. During a collection mission in east and south-east Anatolia (Turkey), a new *Cicer* species was discovered, proposed here as *C. turcicum* Toker, Berger & Gokturk. Here, we describe the morphological characteristics, images, and ecology of the species, and present preliminary evidence of its potential utility for chickpea improvement. *C. turcicum* is an annual species, endemic to southeast Anatolia and to date has only been located in a single population distant from any other known annual *Cicer* species. It belongs to section *Cicer* M. Pop. of the subgenus *Pseudononis* M. Pop. of the genus *Cicer* L. (Fabaceae) and on the basis of internal transcribed spacer (ITS) sequence similarity appears to be a sister species of *C. reticulatum* Ladiz. and *C. echinospermum* P.H. Davis, both of which are inter-fertile with domestic chickpea (*C. arietinum* L.). With the addition of *C. turcicum*, the genus *Cicer* now comprises 10 annual and 36 perennial species. As a preliminary evaluation of its potential for chickpea improvement two accessions of *C. turcicum* were field screened for reproductive heat tolerance and seeds were tested for bruchid resistance alongside a representative group of wild and domestic annual *Cicer* species. *C. turcicum* expressed the highest heat tolerance and similar bruchid resistance as *C. judaicum* Boiss. and *C. pinnatifidum* Juab. & Spach, neither of which are in the primary genepool of domestic chickpea. Given that *C. arietinum* and *C. reticulatum* returned the lowest and the second lowest tolerance and resistance scores, *C. turcicum* may hold much potential for chickpea improvement if its close relatedness supports interspecific hybridization with the cultigen. Crossing experiments are currently underway to explore this question.

## Highlights

– We found that a new species endemic to East Anatolia, Turkey, which we have described and illustrated.
– The new species belongs to the same group with *C. arietinum* L., *C. reticulatum* Ladiz., and *C. echinospermum* P.H. Davis in the genus *Cicer* L. (Fabaceae) according to ITS sequencing.
– Based on preliminary studies, *C. turcicum* is tolerant to some abiotic and biotic stresses including heat, and bruchid that could be used in interspecific crosses to improve domesticated chickpea.

## Introduction

The genus *Cicer* L. has a Rand Distribution, with a center of diversity scattered around the fringes of Africa as the continent has dried over the past few million years (Pokorny et al., 2015). *Cicer* species are from the Atlas Mountains and Canary Islands, in the Ethiopian highlands, to the Balkans and Caucasia, and into South and Central Asia. The richest density of *Cicer* species occur in the Anatolia-Turanian phytogeographic region (van der Maesen, 1972). The genus, despite earlier classifying in the tribe Vicieae Alefeld (1859), has been classified in its own tribe, Cicereae Alef. (Kupicha, 1977; Nozzolillo, 1985; van der Maesen, 1987). In a *Cicer* monograph, van der Maesen (1972) recognized 39 *Cicer* species including 31 perennials and eight annuals, including domesticated chickpea (*Cicer arietinum* L.). Since 1972 to 2007, the following *Cicer* species including *C. heterophyllum* Contandr., Pamukc. & Quezel (Contandriopoulos et al., 1972) from Mediterranean region of Turkey, *C. reticulatum* Ladiz. (Ladizinsky, 1975) from south eastern Turkey, *C. canariense* A. Santos & G.P. Lewis from the Canary Islands (Santos-Guerra and Lewis, 1986), *C. rassuloviae* Linczevski (Czrepanov, 1981), *C. laetum* Rassulova & Sharipova (Rassulova and Sharipova, 1992), and *C. tragacanthoides* Jaubert & Spach var. *turcomanicum* Popov from Turanian region were added to the genus (van der Maesen, 1984; van der Maesen et al., 2007). *C. uludereensis* Donmez (2011), *C. floribundum* Fenzl. var. *amanicola* M. Ozturk & A. Duran, *C. heterophyllum* Contandr., Pamukc. & Quezel var. *kassianum* M. Ozturk & A. Duran and *C. incisum* (Willd.) K. Maly subsp. *serpentinica* M. Ozturk & A. Duran were more recently added as new perennial *Cicer* taxa (Ozturk et al., 2011, 2013). Throughout that period only a single new annual wild *Cicer* species was added. *C. reticulatum*, now considered the wild progenitor of domesticated chickpea, was discovered in Dereici, Savur district, Mardin province, Turkey by Ladizinsky (1975). As a result of these discoveries, by 2020 the number of species in the genus *Cicer* was recognized as 45 species with nine annuals and 36 perennials. Importantly, as outlined below, only two of previously known eight annual wild *Cicer* species (*C. reticulatum* and *C. echinospermum* P.H. Davis) are in the primary and secondary gene pools of cultivated chickpea and are readily inter-fertile with chickpea (Ladizinsky and Adler, 1976a; van der Maesen et al., 2007; Smykal et al., 2015).

Among the annual *Cicer* species, *C. arietinum* is the sole species under domestication and worldwide grown in 60 countries with production quantity of 17.2 million tons from an area of 17.8 million ha in 2018 (FAOSTAT, 2020). Domesticated chickpeas with two varietal groups such as *desi* having pigmented plants, flowers and seeds and *kabuli* having non-pigmented plants, flowers and seeds were mainly grown in Indian sub-continued and Mediterranean region, respectively (Penmetsa et al., 2016). They are a significant source of protein, carbohydrates, vitamins, minerals and unsaturated fatty acids. Chickpeas not only possess characteristics for a balanced diet, especially for poor populations throughout the world (Jukanti et al., 2012; Pradhan et al., 2014; Upadhyaya et al., 2016; Jimenez-Lopez et al., 2020; Sab et al., 2020), but are also important for sustainable agriculture since fixing atmospheric nitrogen to soil via special bacteria provides rotational value to subsequent crops (Afonso-Grunz et al., 2014; Marques et al., 2020b). With climate change, the continued importance of chickpeas depends on their capacity to adapt to adverse environments (Roorkiwal et al., 2014; Ahmad et al., 2016; Deokar and Tar’an, 2016; Pang et al., 2017; Marques et al., 2020a). Gross production value of domesticated chickpea in 2016 has been estimated to be about 5.9 billion $ in the world (FAOSTAT, 2020).

Germplasm resources of annual *Cicer* are not only very limited when compared to cereals and other important food legumes (Berger et al., 2003; Smykal et al., 2015; Foyer et al., 2016; Dwivedi et al., 2019) but also some include several endemic species with endangered status based on the criteria of the International Union for Conservation of Nature (Ozturk, 2011; Talip et al., 2018; Tekin et al., 2018). This is very relevant for chickpea, given the limited diversity of the cultigen, and the ongoing need for new sources of diversity to exploit in crop improvement (Abbo et al., 2003). Currently only two annual *Cicer* species (*C. reticulatum* and *C. echinospermum*) are crossable to cultivated chickpea, but domesticated chickpea is not crossable with other species in the tertiary genepool including *C. bijugum* K.H. Rech., *C. chorassanicum* (Bge) Popov, *C. cuneatum* Hochst. ex Rich, *C. echinospermum, C. judaicum* Boiss., *C. pinnatifidum* Jaub. & Spach, *C. reticulatum*, and *C. yamashitae* Kitamura (van der Maesen et al., 2007). *C. bijugum, C. echinospermum, C. pinnatifidum*, and *C. reticulatum* are native species of Anatolia and Middle-Eastern regions, while *C. cuneatum* occurs in Ethiopia, south-east of Egypt, north of Sudan and Saudi Arabia, *C. chorassanicum* and *C. yamashitae* are distributed to north and north-east of Iran and Afghanistan, and *C. judaicum* is grown in Middle-Eastern region (Robertson et al., 1995; Berger et al., 2003). While *C. judaicum* was incorrectly listed in Turkey (Robertson et al., 1995), only *C. bijugum, C. echinospermum, C. pinnatifidum*, and *C. reticulatum* have been found in Anatolia, Turkey (Davis, 1970; van der Maesen, 1972; Berger et al., 2003; Ozturk, 2011; Ozturk et al., 2011).

**FIGURE 1:**
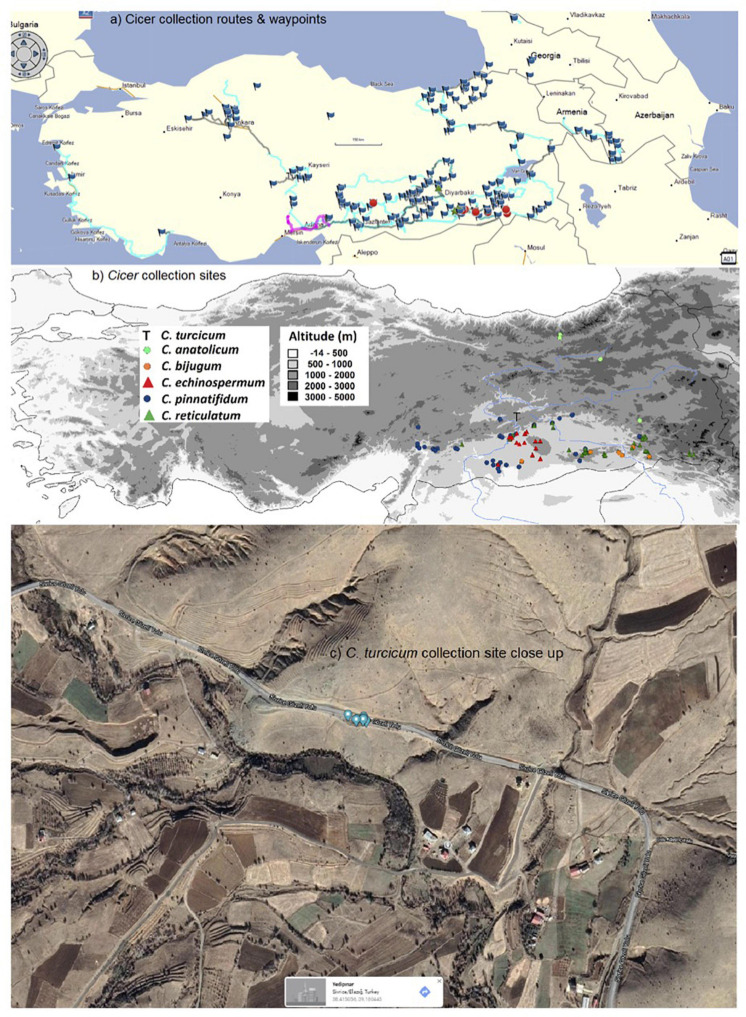
West Asian *Cicer* survey (2013–18) routes and waypoints **(a)**, collection sites classified by species **(b)**, and close-up of the sole *C. turcicum* collection site at Yedipinar collection site, Sivrice district, Elazig province, Turkey. (Image from Google maps, Map data @2021, Australia) **(c)**.

In an effort to expand on these limited crop wild relative resources for chickpea, a *Cicer* collection mission focusing particularly on *C. echinospermum* and *C. reticulatum* was undertaken largely in south-eastern and eastern Turkey from 2013 to 2015 (Toker et al., 2014; Berger et al., 2017, 2018; von Wettberg et al., 2018), with opportunistic side trips from 2016 to 2018 (Figure 1). During the collection mission, *ca* 590 accessions of *C. bijugum, C. echinospermum, C. pinnatifidum*, and *C. reticulatum* were collected from 91 sites and partially evaluated for their adaptive traits (Kahraman et al., 2017; Talip et al., 2018; von Wettberg et al., 2018; Reen et al., 2019; Berger et al., 2020; Newman et al., 2020). This mission covered a huge range of locations throughout Turkey and beyond and collected a new species thus far unknown to the scientific world at only a single site (Figure 1). In the present study we propose this new species as *C. turcicum*, describe its known distribution and ecology, its morphological characteristics and relatedness to other *Cicer* species using internal transcribed spacer (ITS) sequencing. Finally, we undertake a preliminary evaluation for its utility for chickpea improvement by studying the species tolerance to heat in the reproductive stage and seed resistance to the bruchid, *Callosobruchus chinensis* L.

## Materials and Methods

### *Cicer* Survey and Collection Missions

*Cicer* survey and collection missions were conducted from 2013 to 2018 focusing largely on eastern and south-eastern Anatolia with opportunistic side trips through central and western Turkey, southern Armenia, central and western Georgia (Figure 1a). Populations were surveyed in early spring so that plants could be identified using floral characteristics. This entailed random survey of potential collection sites (see waypoints in Figure 1a) by 1–5 scientists searching for any wild *Cicer* species, with a focus on *C. echinospermum* and *C. reticulatum*, and opportunistically recording the presence of any wild *Lens* and *Pisum* relatives (Smýkal et al., 2018). *Cicer* leaf material was collected on a single plant basis to facilitate genetic studies, all samples being individually geo-referenced using a Garmin Montana 650 (von Wettberg et al., 2018). Geo-referenced soil samples were also taken at this time. Mature seeds were collected on an individual plant basis in late spring/early summer and geo-referenced as before.

### Collection Site Climatic Data

Collection site climate data (altitude, monthly mean, minimum and maximum temperature, and precipitation) was extracted at 30 s resolution (ca. 1 km grid) from WorldClim (^1^ Hijmans et al., 2005). Additional climate descriptors (monthly mean frost days, rain days, precipitation coefficients of variance, relative humidity, sun hours, wind speed) were extracted at 10 min resolution (ca. 12 km grid) (^2^ New et al., 2002). Similar climate data were also extracted directly from the weather station at the Elazig airport (892 m asl), 24.1 km distant from the Yedipinar collection site (1,548 m asl) and at lower elevation (892 vs. 1,548 m), courtesy of the Turkish State Meteorological Service (TSMS, 2020).

Site-specific bioclimatic variables such vegetative and reproductive phase rainfall were calculated from these data by defining when plants typically emerged, flowered and matured at each collection site using observation and local feedback crosschecked against seasonal rules imposed on the monthly climate data, and details were given by Upadhyaya et al. (2011).

### Identification

*Cicer turcicum* specimens were compared with the species with the closest resemblance (*Cicer pinnatifidum* and *C. judaicum*) and with specimens at Akdeniz University herbarium. All parts of the specimens were recorded using a ruler with 0.5 mm precision. Photographs were taken with a Sony Alpha 700 digital camera.

### Taxonomic Treatment

According to results of the assessment of morphological including description and habitat with its ecology and molecular data on ITS sequences, the new species were taxonomically classified and evaluated. Also, it was compared to the related species including *C. pinnatifidum, C. judaicum, C. echinospermum*, and *C. reticulatum*.

### Conservation Status

Conservation status was suggested according to plant population and the IUCN threat category (IUCN, 2014).

### DNA Extraction, PCR, and Sequencing

*Cicer arietinum* (ILC 8262 and ICC 8617), *C. reticulatum* (AWC 602), *C. turcicum*, *C. pinnatifidum* (AWC 503 and AWC 505), *C. judaicum* (PI 458559) and *C. cuneatum* were grown under controlled conditions in greenhouse for molecular analysis.

Fresh leaves were stored at –20°C until DNA extraction. Total genomic DNA was extracted using the CTAB method of Doyle and Doyle (1990). DNA concentrations were estimated on 1% agarose gels stained with ethidium bromide. For this study, the nuclear ribosomal internal transcribed spacer region (ITS1, 5.8S rDNA and ITS2) was used to evaluate the relationships between species. The ITS region was amplified using primers ITS 4 and ITS 5 (White et al., 1990). The PCR analysis was carried out with 1 U of *Taq* DNA polymerase (Fermentas Life Sciences, Burlington, ON, Canada) in the supplied reaction buffer, 2 mM MgCl_2_, 0.2 mM of each dNTP, 0.4 μM of each primer and 40 ng of template DNA, and ddH_2_O to a final volume of 15 μL. PCR amplification conditions were as follows: an initial pre-denaturation step at 94°C for 5 min, 35 cycles of 1 min at 94°C, 1 min at 50°C, 1 min at 72°C, and a final extension step of 10 min at 72°C. Amplification was performed on a Bioneer thermocycler (MyGenie^TM^). PCR products were electrophoresed on a 1.5% agarose gel run at 75 V in 1 × TAE buffer and visualized under UV light after staining with ethidium bromide. Sequencing was carried out at Macrogen Inc., Europe via BM Laboratories Ltd., with direct sequencing in both directions using the amplification primers. All sequences were manually edited using Chromas v. 2.6.5 (McCarthy, 1996–1998) and aligned in Bioedit v. 7.0.5.3 (Hall, 1999). Double peaks were represented by IUPAC ambiguity codes in the species of *C. turcicum* in the alignment. Sequences were submitted to GenBank.

### Screening for Heat Tolerance

*Cicer turcicum* phenology and heat tolerance was compared against a range of wild and domestic *Cicer* accessions (Table 1) in a common garden experiment at the Akdeniz University campus Antalya, Turkey (30° 44′ E, 36° 52′ N, 51 m asl). The experiment was conducted in a screenhouse, with plants sown directly into the loam soil for 2 years from 2018–2019 to 2019–2020. Soil properties were given by Kivrak et al. (2020). Water holding capacity, organic matter, soil nitrogen, zinc, and iron were determined to be at low levels, CaCO_3_ and pH were, 26.5 and 7.69%. The experimental design was RCBD with three replications using plots 2 m in length with row spacing of 100 cm, sown on 27th December 2018 in the first year and 29th December 2019 in the second year in order to expose the plants to heat stress during their reproductive phase. Plant phenology (flowering, podding, maturity) was observed at 2–3 day intervals and accessions screened for heat tolerance using a visual 1–9 scale at podset (Table 2). Plants were irrigated with drip irrigation system at 3-day intervals in order to prevent the confounding effects of drought.

**TABLE 1:** Germplasm evaluated for bruchid (B) resistance and heat tolerance (H) experiments (Exp) at Akdeniz University.

Species	Accession ID	Origin	Collection site	Latitude	Longitude	Notes	References	Exp
*C. arietinum*	ACC 100	Tur	Akdeniz Uni			Kabuli, bruchid susceptible check	Eker et al., 2018	B
*C. arietinum*	ACC 1054	Tur	Akdeniz Uni			Kabuli check, heat-sensitive check, cold tolerant	Ceylan et al., 2019	H
*C. arietinum*	ILC 8262	Esp				Kabuli check, heat-sensitive check, cold tolerant	Singh et al., 1992; Canci and Toker, 2009a	H
*C. arietinum*	ILC 8617	ICARDA	Rabat			Kabuli check, heat-sensitive check, cold tolerant	Singh et al., 1995; Canci and Toker, 2009a	H
*C. judaicum*	PI 458559	Pal	Beit Sira, Ramla	32.00	34.83	Wild check,	Robertson et al., 1995; Canci and Toker, 2009a	H, B
*C. pinnatifidum*	IG 72984	Tur	Gaziantep	37.05	37.25	Wild check,	Toker, 2005; Canci and Toker, 2009a	H, B
*C. pinnatifidum*	IG 72986	Tur	Gaziantep	37.05	37.25	Wild check,	Toker, 2005; Canci and Toker, 2009a	H
*C. reticulatum*	IG 72971	Tur	Savur, Mardin	37.30	40.73	Wild check,	Robertson et al., 1995; Canci and Toker, 2009a	H
*C. turcicum*	AWC 404	Tur	Yedipinar, Elazig	38.42	39.18	New annual *Cicer* species		H, B
*C. turcicum*	AWC 551	Tur	Yedipinar, Elazig	38.42	39.18	New annual *Cicer* species		H, B

**TABLE 2:** A visual quantitative 1–9 scale for resistance to a/biotic stresses evaluated in Exps 1 and 2.

Scale	Reaction category	Heat tolerance	Resistance to bruchid
1	Very highly resistant	Very good vigor and 100% pod setting and filling	Damage incidence is 0% and no holes observed
2	Highly resistant	Good vigor and 96–99% pod filling	Damage incidence is about 2–5%
3	Resistant	Good vigor and 86–95% pod filling	Damage incidence is 6–10%
4	Moderately resistant	Moderate vigor and 76–85% pod filling	Damage incidence is 11–20%
5	Moderate	Poor vigor and 51–75% pod filling	Damage incidence is 21–30%
6	Moderately susceptible	Lack of vigor and 26–50% pod filling	Damage incidence is 31–40%
7	Susceptible	Lack of vigor and 11–25% pod filling	Damage incidence is 41–50%
8	Highly susceptible	Lack of vigor and 1–10% pod filling	Damage incidence is 51–90%
9	Very highly susceptible	No flowering or podding	Damage incidence is more than 91%

### Screening for Resistance to the Bruchid

*Callosobruchus chinensis* L. maintained at the Department of Plant Protection, Akdeniz University, Antalya, Turkey were used in a no-choice test after Erler et al. (2009) and Eker et al. (2018). Insect rearing was carried out with susceptible chickpea seeds at 26 ± 2°C and 65 ± 5% RH in complete darkness. To rear fresh adults of a uniform age, seeds with eggs were put in clean jars filled with a large number of chickpea seeds which were checked every day for insect health.

Ten seeds of an accession each of *C. arietinum, C. pinnatifidum*, *C. judaicum*, and two accessions of *C. turcicum* (Table 1) were placed in a separate glass jar of one liter. For each accession, three replications were used. Ten pairs (10♀ and 10♂) of day-old adults of the brıchid were put into each jar. Then glass jars were covered with a gauze cloth in order to anticipate the flight of the insects and to allow air circulation. The bruchids were forced to feed only the seeds of one accession in a jar. After a week oviposition, the adult insects were carefully removed from each jar. Oviposition in each jar was controlled using a stereo-microscope and number of eggs laid by the insect were counted for each accession separately. The jars were controlled daily for adult emergence for 30 days.

Assessment for resistance to the pulse bruchid was evaluated by recording number of eggs per seed, number of holes per seed, percentage of seed damage and seed weight loss in each accession in no-choice test. The number of eggs per seed was recorded with the stereo-microscope. The number of holes was assessed by the round holes with the “flap” on seed coat. Percentage of seed damage was counted as the damaged seeds for each accession, and then data were converted into percentage as damage incidence according to Khattak et al. (1995):

Damageincidence(%)=(Noofseedsdamaged/Totalnoofseeds)×100

The damage incidence was classified according to Table 2. A similar scale in *Cicer* and *Pisum* species was successfully used by Eker et al. (2018) and Esen et al. (2019), respectively. Seed weight loss was determined the following formula (Khattak et al., 1995):

$$Totalloss\left(\%\right)=\left(n_{2}-n_{1}\right)/n_{2}100,$$

where *n*_2_ and *n*_1_ are the initial weight of seeds before the test and the weight of the damaged seeds after the test, respectively.

### Data Analyses

The phylogenetic tree was constructed with the Maximum Parsimony (MP) method using MEGA v. 7 (Kumar et al., 2018), under heuristic searches with 100 random addition sequence replicates and tree-bisection-reconnection (TBR) branch swapping, saving no more than 100 trees with length ≥ 1 per replicate, automatically increasing the maximum number of trees saved. Bootstrapping was performed using the same settings and 1,000 replicates, but without branch swapping. For the phylogenetic analyses, available sequences of *C. echinospermum* (AB198910.1) and *C. bijugum* (AJ237701.1) were retrieved from GenBank for comparison. Also, the sequences data belongs to *P. sativum* L. (L36637.1) and *L. culinaris* Medik. subsp. *orientalis* (Boiss.) Ponert (AJ441321.1) were used as outgroups in the phylogenetic analyses.

Visual scale data were converted to percentage and then used to perform analysis of variance (ANOVA) using Genstat V20 software, nesting accessions within species. Residual plots were generated to detect errors and confirm common and independent variance. For each stressor, significant differences between the accessions were studied using LSD and Duncan multiple range tests.

## Results

### Collection of *C. turcicum*

**FIGURE 2:**
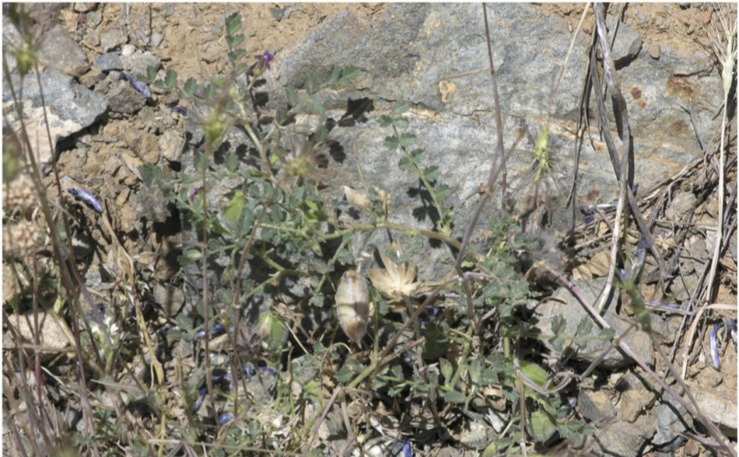
*C. turcicum in situ* at Yedipinar collection site, Sivrice district, Elazig province, Turkey on 12th June 2015. Specimen is both flowering and podding, with some mature pods.

Plant specimens and mature seeds were collected near Yedipinar collection site in the Sivrice district (Elazig Province, Turkey) on 12th June 2015 (Figure 1). Plants were flowering and podding, with some mature pods (Figure 2).

*Cicer turcicum* appears to be a rare species, found only once among the 242 sites surveyed in Turkey, Armenia and Georgia (Table 3). The Yedipinar collection site is remote from other known occurrence of annual wild *Cicer* (Figure 1b), 38 km from the closest *C. pinnatifidum*, 46 km from *C. reticulatum*, 49 km from *C. echinospermum*, and 124 km from *C. bijugum*.

**TABLE 3:** Number of survey sites in which wild *Cicer* species were found, categorized by country and species.

Country	Armenia	Georgia	Turkey	Total
Total survey sites	25	8	209	242
**Annual wild *Cicer* species**				
*C. reticulatum*			40	40
*C. echinospermum*			18	18
*C. bijugum*			7	7
*C. pinnatifidum*			38	38
*C. turcicum*			1	1
**Perennial wild *Cicer* species**				
*C. anatolicum*	1		4	5
*C. isaricum*			1	1

### Species Biology and Habitat Characterization

*Cicer turcicum* is an East Anatolian endemic in the Irano-Turanian phytogeographic region. Habitat is a hilly area with some trees cover on the slopes ranging from isolated oak woodlands, oak/juniper forest and some pine plantations. The plants were located in a tight cluster in a light brown sandy loam on a S-facing rubble slope adjacent to the Sivrice-Gozeli road (38.4174N, 39.1783E) in moderately dense annual vegetation at 1,544–1,553 m elevation (Figure 1c).

**FIGURE 3:**
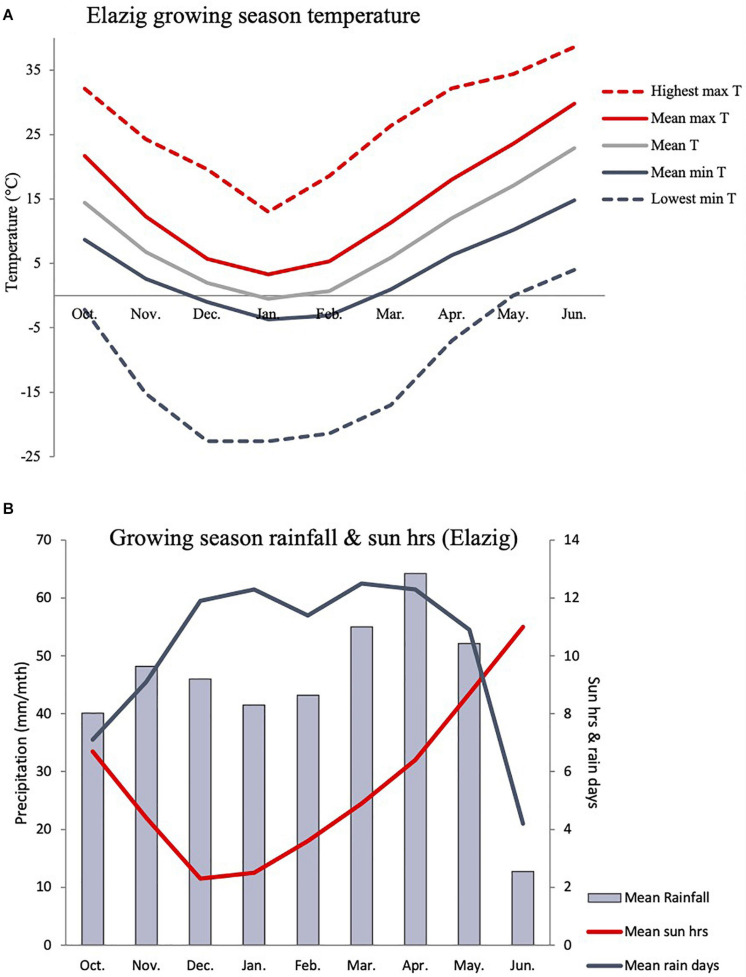
Indicative *C. turcicum* growing season temperature **(A)**, precipitation and sun hours **(B)** based on long term monthly data (1981–2010) from the Elazig airport weather station (892 m asl), located 24.1 km from the Yedipinar collection site (1,548 m asl) at lower elevation (892 vs. 1,548 m). *C. turcicum* germinates in October, flowers in April/May and matures in June/July based on field observations and phenology data from common garden evaluation (see Figure 6).

Based on *in-situ* field observation made during the *Cicer* survey and collection mission, *C. turcicum* phenology seems most similar to *C. reticulatum* and somewhat later than *C. pinnatifidum.* Most of the latter species observed close to the Yedipinar collection site had mature, shattered pods at this time (see also subsequent phenology data from common garden comparison). On this basis we expect *C. turcicum* to germinate with the opening autumn rains (October), start flowering in late April/early May and mature from mid-June onward like other annual *Cicer* species. Climate at the collection site of *C. turcicum* is typically Mediterranean, with arid summers and cold winters. The area is relatively cool, with snow cover 5 months of the year, reflecting the relatively high elevation (Table 4). Accordingly, *C. turcicum* receives most of its seasonal rainfall during the vegetative phase, characterized by frequent, reliable precipitation, high relative humidity, and low sunshine (Table 4 and Figures 3A,B). Vegetative mean temperatures are very low and there is a high incidence of frost (Table 4). The mean reproductive phase climate is mild, with relatively low temperatures, a low rate of temperature increase, and relatively frequent rainfall (Table 4). Monthly mean temperatures from climate databases do not capture the climatic extremes that are likely to exert strong selection pressure on endemic plant species. This is demonstrated by data from the nearby (albeit considerably lower altitude) Elazig airport weather station which shows that temperatures can range from <−20°C to >40°C in the vegetative and reproductive phases, respectively (Figure 3A). Given the much greater elevation of the Yedipinar collection site, it is likely that minimum temperatures may range even lower than this, while reproductive phase temperatures may not be as extreme.

**TABLE 4:** Characteristics of the sole *C. turcicum* collection site at Yedipinar village, Sivrice district, Elazig province, Turkey based on geographic data extracted from Garmin Montana 650 and climate data from WorldClim (Hijmans et al., 2005) and 10 min Climatology (New et al., 2002), calculated over the indicated growing season phases.

Descriptor	Site mean	Pre-season (July-Oct)	Veg phase (Oct-May)	Rep phase (May-June)	Season total
Latitude (°d)	38.4175				
Longitude (°d)	39.1782				
Elevation (m)	1548				
Mean temp (°C)			3.9	17.1	6.8
Min temp (°C)			−6.6		
Max temp (°C)				26.0	
Temp change (°C/day)				0.1	
Frost days (sum)			94	1	94
Precipitation (sum, mm)		15	554	88	642
Precipitation CV (%)			59	81	64
Rain days (sum)			82	17	98
Rel humidity (%)			66	44	61
Sun hrs (%/day)			52	75	58

### Taxonomy-Morphology of *C. turcicum*

#### Description

**FIGURE 4:**
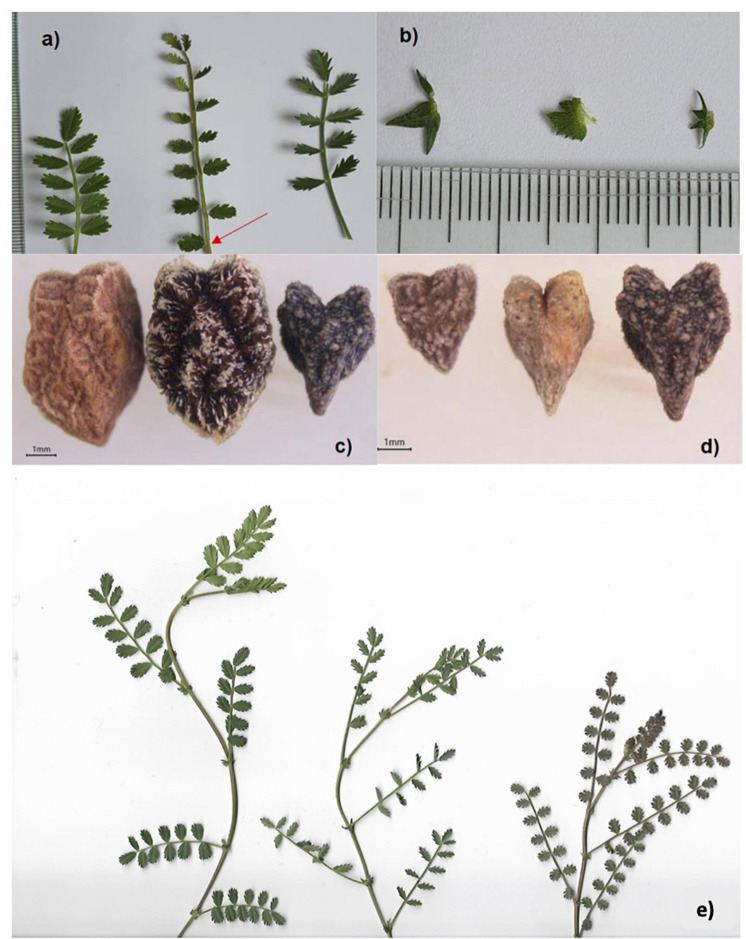
Leaves of *C. pinnatifidum*, *C. turcicum* and *C. judaicum* (**a**, left to right). Single leaflet at the base of leaves of *C. turcicum* (red arrow). Stipules of *C. judaicum*, *C. pinnatifidum*, and *C. turcicum* (**b**, left to right). Seeds of *C. reticulatum*, *C. echinospermum*, and *C. turcicum* (**c**, left to right). Seeds of *C. judaicum*, *C. pinnatifidum*, and *C. turcicum* (**d**, left to right). Shoots of *C. pinnatifidum*, *C. judaicum*, and *C. turcicum* (**e**, left to right).

Annual; stem semi-prostrate up to 45 cm long, procumbent branches at base, completely pubescent, glandular hairs. Leaves imparipinnate with 7 pairs of leaflets; rachis 3–5 × 0.7–1.1 cm in outlines; petiol 5–6 mm; leaflets pubescent, fairly close, opposite or not, shortly petiolulate 0.5 mm, oblong-elliptic, 5–6 (−7) × 2–4 mm, and a single leaflet at base of rachis (arrow in Figures 4a,e), basal 1/5 part entire; teeth 9–11 (−14), acute. Stipules pubescent, four unequal teeth, each teeth triangular, 2–3 × 4–6 mm (Figure 4b). Inflorescence generally 1-flowered (seldom double-flowered), axillary racemes; peduncle pubescent, 4–10 mm, ending in an arista, 2 mm; bracts linear, 0.5 mm; pedicel pubescent, 4–8 mm. Calyx hardly dorsally gibbous at the base, pubescent, 3–6 mm, teeth triangular-lanceolate, 2–4 mm. Corolla veined, glabrous, purple-magenta, fading into blue-violet and magentaroadly ovate, when old; standard (wexillum), emarginated at apex, attenuate at base, 8–10 × 6–8 mm; wings (alae) obovate, strongly auriculate at base, 6–7 × 2–3 mm; keel (carina) rhomboid, 4–5 × 1.5–2.5 mm. Stamens diadelf (9+1), filaments 5–6 mm long (fused part 4 mm, free part 1.5–2 mm, upturned). Ovary ovoid, 6 mm long, densely glandular pubescent; style ca. 2–4 mm, upturned. Pods rectangular ovate at base, 15–18 × 6–9 mm, stylus and stamens persistent when old, 3–4 seeds, shattered when ripe. Seeds triangular-arietinoid, distinctly bilobular, beaked, 5–6 × 4–5 mm, hilum 0.5–1 mm, seed coat greenish-dark brown, tuberculated (Figures 4c,d).

*Cicer turcicum* is completely different from *C. pinnatifidum, C. judaicum, C. echinospermum*, and *C. reticulatum* because of gross morphology and seed size/shape differences (Figure 4 and Table 5). Flowers, pods and seeds of the new species are larger than those of *C. pinnatifidum* and *C. judaicum*, while they were smaller than those of *C. echinospermum* and *C. reticulatum* (Table 5). The new species can easily be distinguished by differences in leaflets (one of leaflets at the base of leaf is single), stipules (Crown-shaped), and seeds (greenish-dark brown and tuberculate) from *C. pinnatifidum, C. judaicum, C. echinospermum*, and *C. reticulatum* (Figure 4).

**TABLE 5:** Comparison of *C. turcicum* for diagnostic characteristics with *C. pinnatifidum, C. judaicum C. echinospermum*, and *C. reticulatum*.

Characteristics	C. judaicum*	C. pinnatifidum*	*C. turcicum*	C. echinospermum*	C. reticulatum***
Leaves (no)	(7–9) 11–13	4–9 (11)	13–14	7–11	8–15
Leaflets (mm)	4–7 (9) × 2–5 (8)	4–11 (12) × 2–5 (7)	5–6 (7) × 2–4	4–9 (11) × 2–5	5–11 (15) × 2–4
Stipules (no of teeth)	3	6–7	4	3–5	4–5
Seeds (mm)	3–4 × 3–4	4–6 × 3–5	5–6 × 4–5	7 × 5	5–9 × 4–6
Pods (mm)	10–13 × 5–6	10–15 × 6–8	15–18 × 6–9	15–20 × 10–12	12–16 × 8–12
Leaflet position	Opposite or not	Opposite or not	Opposite or not but a single leaflet at base	Opposite or not	Opposite or not
Distribution**	Levant (Isr, Pal, Leb, Syr)	Levant, S & SE Anatolia	E Anatolia (1 location)	SE Anatolia	SE Anatolia

*, **, and *** Data were obtained by van der Maesen (1972); Berger et al. (2003), and Ladizinsky (1975), respectively.

#### Taxonomic Treatment

Based on morphological and molecular data allowing comparison to the related samples, it was decided that the specimens collected from Elazig belongs to a new species. This species was named *C. turcicum* and taxonomically put in subgenus *Pseudononis* M. Pop. and section *Cicer* M. Pop. (van der Maesen et al., 2007; Ozturk et al., 2013).

*Cicer turcicum* Toker, Berger & Gokturk, sp. nov. (Figures 4a–d).

Type: —TURKEY. B7 Elazig: Sivrice, Yedipinar around (38.4174N, 39.1783E) at 1,544–1,553 m elevation, in June 2015, Toker, Berger (1001) & Gokturk (holotype Akdeniz University herbarium!, isotypes PAMUH!, ANK!, HUB!, GAZI!).

#### Etymology

The specific epithet is derived from the name of Turkey.

#### Alignment and Sequence Characteristics

Nucleotide sequences were deposited in GenBank (accessions MW424513-MW424518). The ITS region (ITS1-5.8S gene-ITS2) in *Cicer* ranged from 692 to 704 bp. The aligned length for the ITS dataset was 662 positions, with 58 informative sites and 118 variable sites. In total, 43 diagnostic single nucleotide polymorphisms and one tri-nucleotide deletion were observed in the aligned dataset. No intraspecific variation was observed in *C. arietinum*, *C. turcicum*, and *C. pinnatifidum. C. turcicum* showed seven single nucleotide identities to the sequences of *C. arietinum, C. reticulatum*, and *C. echinospermum* (Table 6), positions: (45, 78, 103, 204, 457, 472, 474). This species had an identical nucleotide with *C. pinnatifidum* in the position of 98. Additionally, their three nucleotide deletions (GAC, position: 205–207) were shared. *C. turcicum* had double peaks in direct sequences, so additive characters were represented in the positions of 536 and 588 (Table 6). These characters were not observed in any other species.

**TABLE 6:** Species diagnostic differences in ITS region.

Species	Position in alignment										
	45	78	98	103	204	205–207	457	472	474	536	588
*C. arietinum*	A	C	A	C	T	GAC	G	C	T	T	A
*C. reticulatum*	A	C	A	C	T	GAC	G	C	T	T	A
*C. echinospermum*	A	C	A	C	T	GAC	G	C	T	T	A
*C. turcicum*	A	C	G	C	T	GAC	G	C	T	Y*	R*
*C. judaicum*	C	T	A	–	G	–	A	T	C	T	A
*C. pinnatifidum*	C	T	G	–	G	–	A	T	C	T	A
*C. cuneatum*	C	T	A	–	G	–	A	T	C	T	A
*C. bijugum*	C	T	A	–	G	–	A	T	C	T	A

*Additive characters (double peaks in direct sequences) are represented by IUPAC codes in bold.

#### Phylogenetic Analysis of the ITS Region

**FIGURE 5:**
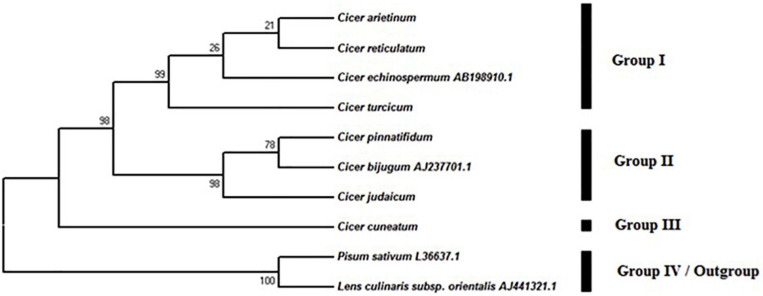
Phylogenetic tree from the maximum parsimony analysis based on the sequence of ITS region in *Cicer* taxa.

The MP analysis resulted in 10 equally parsimonious trees (length: 133) with a consistency index (CI) of 0.898, a retention index (RI) of 0.917 and a rescaled index (RC) of 0.868. In the phylogenetic tree, four major groups were observed (Figure 5), one of which included *P. sativum* and *L. culinaris* subsp. *orientalis* as out-group, while the rest were taxa in the genus *Cicer*. Group I, consisting of *C. arietinum, C. reticulatum, C. echinospermum*, and *C. turcicum*, was supported by a 98% bootstrap value in the parsimony tree. This group revealed two subgroups (Figure 5). There was a strong support that *C. turcicum* was in the different group from *C. arietinum, C. reticulatum, C. echinospermum* (bootstrap support, 99%). Group II included *C. pinnatifidum*, *C. bijugum*, and *C. judaicum*. This group showed a bootstrap value of 98%. Group III only consisted of *C. cuneatum*.

#### Phenology

**FIGURE 6:**
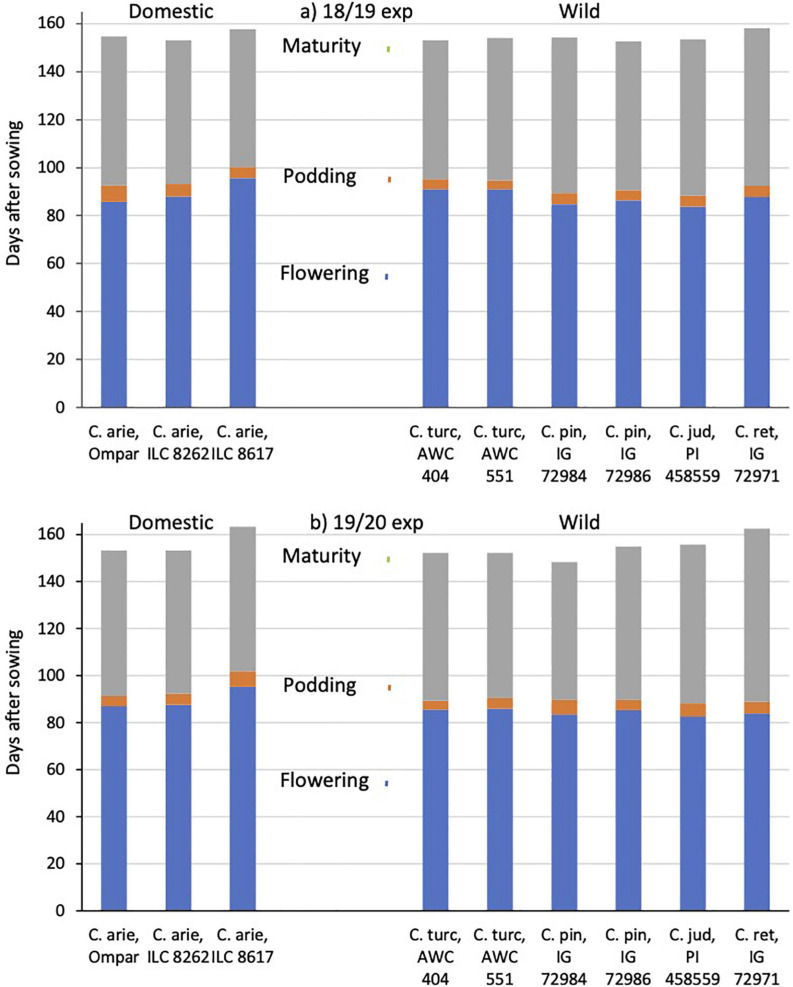
*C. turcicum* phenology (flowering, podding, maturity) compared to related annual wild and domestic *Cicer* species. Data is from Mediterranean cool-season common garden screenhouse comparisons at Akdeniz University, **(a)** 2018/19, **(b)** 2019/20. Color-coded vertical lines represent accession least significant differences (LSD *P* < 0.05) for flowering (2.6–2.7 days), podding (2.4–2.7 days) and maturity (1.9–2.4 days). Abbreviations: C. arie, *C. arietinum*; C. turc, *C. turcicum*; C. pin, *C. pinnatifidum*; C. jud, *C. judaicum*; C. ret, *C. reticulatum*.

Common garden evaluations in the Akdeniz University screenhouse in 2018/19 and 2019 confirmed field observations made at the Yedipinar collection site regarding the typical Mediterranean winter annual phenology of *C. turcicum*. In the 2018/19 experiment, *C. turcicum* flowered and podded slightly later than *C. reticulatum*, followed by the remaining annual wild *Cicer* species, while in the following year there were no significant differences among any of the annual wild *Cicer* species (Figure 6). Domestic chickpea covered a wider range, the cultivar Ompar and ILC 8262 returning intermediate flowering and podding dates, while ILC 8617 was consistently 7–10 days later in both years (Figure 6, *P* < 0.001). *C. turcicum* matured relatively early, particularly in the 2019/20 experiment, where it was earlier than *C. judaicum*, an accession of *C. pinnatifidum* and particularly *C. reticulatum* (Figure 6). In 2018/19, wild *Cicer* maturity was more evenly distributed, with only *C. reticulatum* maturing at a later date than the rest of the group. *C. arietinum* maturity dates followed the other phenological data, Ompar and ILC 8262 maturing early (similar to *C. turcicum*), +while ILC 8617 was consistently 3–10 days later (Figure 6, similar to *C. reticulatum*).

#### Heat Tolerance

**FIGURE 7:**
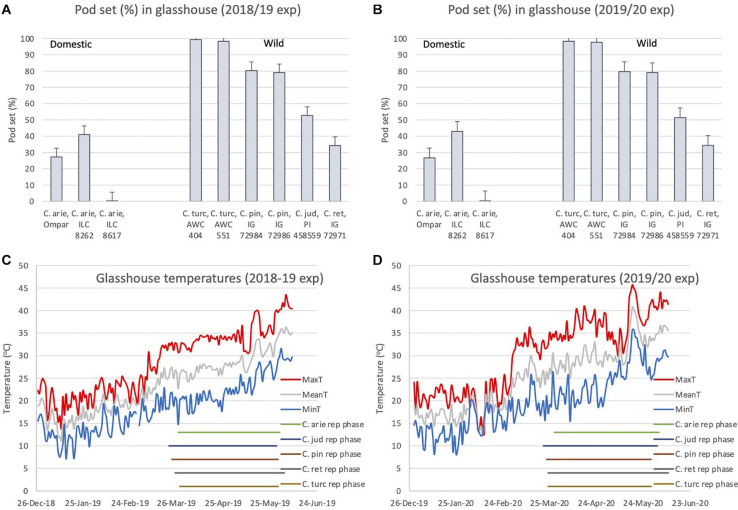
*C. turcicum* pod set percentage **(A,B)** under high reproductive phase temperatures **(C,D)** compared to related annual wild and domestic *Cicer* species. Data is from Mediterranean cool-season common garden screenhouse comparisons at Akdeniz University, **(A,C)** 2018/19, **(B,D)** 2019/20. Error bars represent accession least significant differences (LSD *P* < 0.05). Reproductive phase lengths (flowering to maturity) are shown individually for each species **(B)**. Abbreviations: C. arie, *C. arietinum*; C. turc, *C. turcicum*; C. pin, *C. pinnatifidum*; C. jud, *C. judaicum*; C. ret, *C. reticulatum*.

Despite the broad phenological similarities described above (Figure 6), there were dramatic differences in pod setting under elevated reproductive phase temperatures between wild and domestic *Cicer* species in both years (Figure 7). ANOVA indicated large species differences across years (*P* < 0.001), without interaction (*P* < 0.574), and smaller differences between varieties within species (*P* < 0.001), again without interaction over years. Thus, while pod set percentage means of all wild *Cicer* species were greater than in domestic chickpea (*P* < 0.001), *C. turcicum* > *C. pinnatifidum* > *C. judaicum* > *C. reticulatum* (Figures 7A,B *P* < 0.05). Pod set in domestic chickpea germplasm varied from 0% in ILC 8617 to 43% in ILC 8262, the latter variety setting a greater proportion of pods than IG 72971 (*P* < 0.05), the sole representative of *C. reticulatum* in this experiment. Analysis of the diurnal temperatures ranges recorded during the experiment demonstrated that heat escape resulting from variable phenology was not a factor in these inter-specific differences (Figures 7C,D). Mean temperatures increased linearly throughout the reproductive phase (2018/19, 0.11°C/day, *r*^2^ = 0.71; 2019/20, 0.13°C/day, *r*^2^ = 0.62) from *ca*. 26°C at flowering to > 35°C at maturity (Figures 7C,D). While temperature maxima fluctuated more on a daily basis, with weaker linear trends (2018/19, 0.09°C/day, *r*^2^ = 0.49; 2019/20, 0.10°C/day, *r*^2^ = 0.36), all species experienced maxima > 40°C during podding in both years, and none escaped sharply rising temperatures toward the end of the growing season in either year (Figures 7C,D).

#### Resistance to Bruchid

**FIGURE 8:**
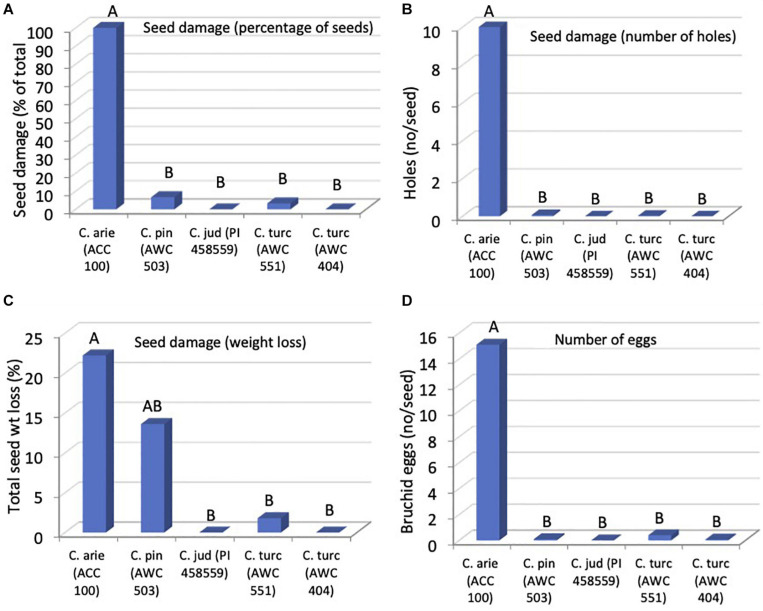
Bruchid resistance in wild compared to domestic *Cicer* species in terms of seed damage **(A)**, number of holes **(B)**, weight loss **(C)**, and number of eggs **(D)**, from a no-choice feeding test at Akdeniz University. Letters represent accession group membership from Duncan multiple range test, different letters indicate significant difference (*P <* 0.05).

Orthogonal contrasts revealed striking wild-domestic differences in bruchid resistance, accounting for all of the significant species differences. Seed damage was far lower in wild compared to domestic *Cicer*, whether measured as the number of holes on the seed coat (Figure 8, *P* < 0.001), percentage of seeds damaged (*P* < 0.001) or in terms of seed dry matter consumed by the bruchids (*P* < 0.031). As a result, bruchid egg production was far lower on wild compared to domestic *Cicer* (Figure 8, *P* < 0.001). There were no significant differences among wild *Cicer* for any of these traits, nor between the two *C. turcicum* accessions evaluated in the present study (*P*_diff_ = 0.452–0.976).

## Discussion

In the present study we introduce *C. turcicum*, a new annual wild *Cicer* species hitherto unknown to science. *C. turcicum* appears to be a rare species, thus far recorded only in a single location in Elazig province, SE Anatolia, at a considerable distance from the nearest known wild *Cicer* population (Figure 1). The Yedipinar collection site has a realtively high elevation (*ca* 1,550 m) and exposes *C. turcicum* to an extreme temperature range throughout the growing season, from very cold winters to hot, dry summers. *C. turcicum* has a distinct morophology that separates it from wild relatives, paticularly leaflet and seed size, distribution and shape (see Figure 4 and Table 5), while ITS sequencing suggests it to be closely related to *C. arietinum*, *C. reticulatum*, and *C. echinospermum* (Figure 5). Common garden evaluation demonstrates that *C. turcicum* has a typical annual wild *Cicer* phenology, but appears to be more tolerant of reproductive heat stress than its wild relatives, and similarly resistant to bruchid feeding.

These findings raise a number of interesting implications and questions that need to be followed up. Arguably the most important of these is species rarity. The 2013–2018 *Cicer* mission surveyed 242 sites in detail, geo-referencing the presence/absence of wild crop legume relatives (*Cicer*, *Pisum*, *Lens*) and noting associated species. The fact that *C. turcicum* was only found at a single location underlines its relative scarcity. However, while the region immediately south of the Yedipinar collection site has been comprehensively surveyed (Figure 1), there were very few sites in Elazig province itself, particularly the areas surrounding Yedipinar to the north. A population of *C. pinnatifidum* was found at Tepekoy, 38 km to the west of Yedipinar, while *Lens* was found between Maden and Ergani, 42 km to the east of Yedipinar. Clearly, there is more work to do to establish the *C. turcicum* distribution. However, at this stage, with only a single collection site identified, it may be prudent to place *C. turcicum* under the IUCN threat category “Critically Endangered (CR)” (IUCN, 2014) because its estimated area of occupancy is less than 10 km^2^, population size is estimated to be less than 50 mature individuals, and is under threat of heavy grazing pressure [CR B2; C2a(i)] given its proximity to Yedipinar and the Sivrice-Gozeli road (Figure 1c). In the meantime, we suggest that further survey missions focusing on Elazig province be undertaken as a matter of urgency.

While the identification of any new species is of in interest in its own right, the fact that *C. turcicum* is both an annual and appears to be closely related to *C. arietinum*, the single domesticated *Cicer* species makes it all the more important because annual *Cicer* species are relatively uncommon and its relatedness to chickpea opens new questions regarding the domestication of this crop. The ITS-sequencing phylogeny presented in this study reflects the current taxonomic status of the species. Thus, *Pisum* and *Lens* were outgroups, reflecting their status as genera in the tribe Fabeae Rchb. referred to as Vicieae (Schaefer et al., 2012), while all the *Cicer* species were broadly clustered in Cicereae (Javadi and Yamaguchi, 2004; Schaefer et al., 2012). The within *Cicer* species clustering closely followed the known genepool (GP) classification:

(1) GP1: *C. arietinum* (domesticated chickpea) and *C. reticulatum* (Ahmad, 1999). Hybridization in the primary gene pool (GP1) is straightforward, progeny are fully fertile due to good chromosome pairing, alien gene transfer is achievable from wild to domesticated chickpea with traditional methods (Ladizinsky and Adler, 1976a,b; Adak et al., 2017; Koseoglu et al., 2017).
(2) GP2: *C. echinospermum*. Species in GP2 can be crossed with domesticated chickpeas and produced at least some fertile progeny, while hybrids are weak, partly sterile, and recovery of progeny in subsequent generations is difficult due to post fertilization problems (Mallikarjuna et al., 2011). Hybridization success varies between accessions (Kahraman et al., 2017). The proximity of *C. turcicum* to *C. echinospermum* in the ITS dendrogram (Figure 5) suggests that it is likely to be a member of GP2. To confirm this a hybridization program crossing *C. turcicum* with *C. arietinum* and *C. echinospermum* should be established.
(3) GP3. Species in GP3 are difficult to cross successfully with domesticated chickpeas (Ahmad et al., 1988; Badami et al., 1997; Clarke et al., 2006; Abbo et al., 2011) and include *C. bijugum, C. judaicum, C. pinnatifidum*, and *C. cuneatum*. Our ITS phylogeny places *C. cuneatum* in a separate cluster from the other GP3 species, and is in agreement with an earlier RAPD-derived phylogeny (Ahmad, 1999).

The discovery of *C. turcicum* at a single location in Yedipinar location, Sivrice district, Elazig province underlines the importance of Turkey as a center of biodiversity, particularly of the wild relatives of domesticated crops including chickpea. Turkey includes over 30% endemic species of approximately 12,000 natural vascular plant taxa in the world including 3,788 endemics (Guner et al., 2012). These are well documented using a grid system (Ture and Bocuk, 2010) and are distributed in different phytogeographical regions that intersect in Anatolia. A total of 17 *Cicer* taxa including domesticated chickpea, *C. anatolicum, C. bijugum, C. echinospermum, C. floribundum var. floribundum, C. floribundum var. amanicola, C. heterophyllum var. heterophyllum, C. heterophyllum var. kassianum, C. insicum subsp. incisum, C. incisum subsp. serpentinica, C. isauricum, C. montbretti, C. pinnatifidum, C. reticulatum, C. oxydon, C. turcicum*, and *C. uludereensis* are known to occur in Anatolia. The distribution of both extant *Cicer* species and their archeological remains suggest that Anatolia is not only the primary gene center of the genus *Cicer*, but also the cradle of the genus in terms of species richness.

Finally, the preliminary discovery of heat tolerance and bruchid resistance in *C. turcicum* add value to it’s role as a donor in crop improvement should it be readily crossable with chickpea, or as experimental material to study responses to these stresses if it is not readily crossable. Heat stress causes yield loss in chickpea: day temperatures > 32°C reduces pod set (Basu et al., 2009). The incidence of heat stress in chickpea is predicted to rise in line with the 2–3°C temperature rise expected as a result of climate change in the near future (IPCC, 2007; Hatfield and Prueger, 2015). Although a number of studies have been carried out on heat tolerance in cultivated chickpea (Canci and Toker, 2009b; Krishnamurthy et al., 2011; Upadhyaya et al., 2011; Devasirvatham et al., 2015; Farooq et al., 2017; Paul et al., 2018) and its wild relatives (Canci and Toker, 2009a) yet they have generally found insufficient variation to meet this challenge. The observed heat tolerance of *C. turcicum* aligns well with the climate of the site of origin, characterized by an extreme temperature range. Note that the evidence for heat tolerance in *C. turcicum* is particularly compelling because the temperature data indicates that all species were subject to the same high reproductive phase temperature range, meaning that there were no heat escape opportunities. Nor is it likely that *C. turcicum* was more tolerant than the remaining wild species because of faster pod set, given that it’s seed size is larger than both *C. pinnatifidum* and *C. judaicum*. Vegetative frost and reproductive chilling tolerance are also sorely lacking in domestic chickpea (Berger et al., 2012). Given, the cold nature of the of *C. turcicum* collection site, it is possible that this species may also harbor useful cold tolerance.

Bruchid resistance is also rare in domestic chickpea. Although more than 3,000 chickpea accessions were evaluated for resistance to *C. chinensis* at the International Center for Agricultural Research in the Dry Areas (ICARDA), no resistance was found in *kabuli* types. However, while some resistant *desi* chickpea with thick, rough or tuberculate seed coats have been identified (Reed et al., 1987), wild species such as *C. echinospermum* were found to be “immune” or free from damage (Eker et al., 2018). Annual *Cicer* species have already been screened for resistance to seed bruchid prior to the present study, and all accessions of *C. echinospermum* (100%), some accessions of *C. bijugum* (42.9%), *C. judaicum* (12.8%), and *C. reticulatum* (5%) were outlined to be free from the insect damage (Singh et al., 1998).

## Conclusion

The following conclusions can be drawn from the present study:

• *C. turcicum* is a new, morphologically and genotypically distinct annual *Cicer* species which appears to be rare and found in different, climatically extreme environments than its *Cicer* relatives.
• ITS sequencing places it within the secondary genepool of domestic chickpea; this needs to be confirmed by crossing studies.
• Preliminary evaluation shows *C. turcicum* to harbor heat tolerance and bruchid resistance, but needs to be confirmed with wider evaluation.

The above list suggests that *C. turcicum* will be useful for chickpea improvement if the species can be successfully crossed with the cultigen, but that it also represents an interesting opportunity for domestication and trait discovery studies if that is not the case. Regardless, *C. turcicum* is rare, and needs better understanding/protection. We suggest further survey and collection focusing on Elazig province in SE Anatolia, and registration in a “Critically Endangered (CR)” IUCN threat category.

## Data Availability Statement

The original contributions presented in the study are publicly available. This data can be found here: Sequences were submitted to GenBank with the accession numbers of AB198910.1.

## Author Contributions

CT, JB, and RSG designed the studies. JB, BA, and AK found the new species. CT and RSG described the new species. DS and HS performed the molecular study. TE and HS conducted heat tolerance and bruchid studies. CT, JB, RSG, and EW wrote and revised the manuscript. All authors contributed to the article and approved the submitted version.

## Conflict of Interest

The authors declare that the research was conducted in the absence of any commercial or financial relationships that could be construed as a potential conflict of interest.

## Footnotes

1 http://www.worldclim.org

2 http://www.cru.uea.ac.uk/cru/data/hrg/
